# A New Strategy to Quantitatively Identify Hot-Spot Areas in Growth of New HIV Infections for Targeted Interventions

**DOI:** 10.3389/fpubh.2021.680867

**Published:** 2021-07-12

**Authors:** Qiyu Zhu, Chunnong JiKe, Chengdong Xu, Shu Liang, Gang Yu, Ju Wang, Lin Xiao, Ping Liu, Meibin Chen, Peng Guan, Zhongfu Liu, Cong Jin

**Affiliations:** ^1^National Center for AIDS/STD Control and Prevention, Chinese Center for Disease Control and Prevention, Beijing, China; ^2^Department of Epidemiology, School of Public Health, China Medical University, Shenyang, China; ^3^Liangshan Prefecture Center for Disease Control and Prevention, Xichang, China; ^4^State Key Laboratory of Resources and Environmental Information System, Institute of Geographic Sciences and Natural Resources Research, Chinese Academy of Sciences, Beijing, China; ^5^Sichuan Provincial Center for Disease Control and Prevention, Chengdu, China

**Keywords:** HIV/AIDS, recent HIV infection, geospatial analysis, geographic distribution, quantitative study

## Abstract

**Background:** Previous geographic studies of HIV infection have usually used prevalence data, which cannot indicate the hot-spot areas of current transmission. To develop quantitative analytic measures for accurately identifying hot-spot areas in growth of new HIV infection, we investigated the geographic distribution features of recent HIV infection and long-term HIV infection using data from a whole-population physical examination in four key counties in Liangshan prefecture, which are most severely affected by HIV in China.

**Methods:** Through a whole-population physical examination during November 2017- June 2018 in the four key counties, a total of 5,555 HIV cases were diagnosed and 246 cases were classified as recently infected by laboratory HIV recency tests. The geospatial patterns of recent and long-term HIV infected cases were compared using ordinary least squares regression and Geodetector. Further, geospatial-heterogeneity was quantified and indicated using a residual map to visualize hot-spot areas where new infection is increasing.

**Results:** The geographic location of HIV cases showed an uneven distribution along major roads and clustered at road intersections. The geographic mapping showed that several areas were clustered with more recently infected HIV cases than long-term infected cases. The quantitative analyses showed that the geospatial asymmetry between recent and long-term HIV infection was 0.30 and 0.31 in ordinary least squares regression and Geodetector analysis, respectively. The quantitative analyses found twenty-three townships showing an increase in the number of recent infections.

**Conclusions:** Quantitative analysis of geospatial-heterogeneous areas by comparing between recent and long-term HIV infections allows accurate identification of hot-spot areas where new infections are expanding, which can be used as a potent methodological tool to guide targeted interventions and curb the spread of the epidemic.

## Introduction

The human immunodeficiency virus (HIV) has caused a severe public health burden globally, with an estimated 37.9 million people living with HIV in 2018 (1). In 2018, there were an estimated 1.25 million people living with HIV in China (2), posing a huge challenge to public health workers to control the epidemic. In many regions, the distribution of the HIV epidemic is uneven, with high prevalence in some areas and populations. In this context, targeted interventions are important to achieve effective HIV epidemic control, which has been shown to maximize the effect of prevention at the lowest cost (3). Therefore, knowing where and whom new infections occur is critical for informing targeted interventions.

**Figure 1:**
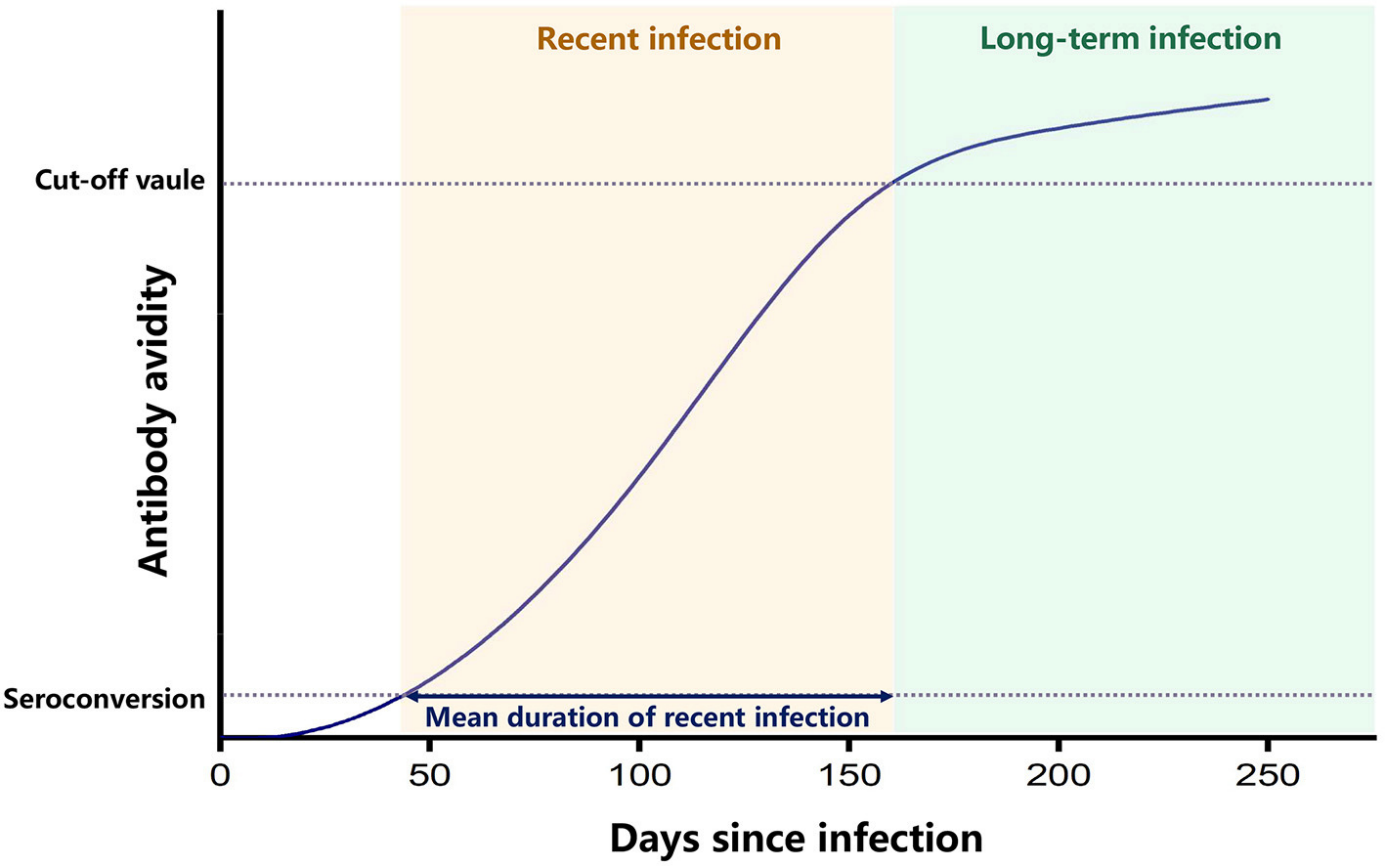
Principle of LAg-EIA. This graph indicates that after seroconvertion, the avidity of HIV-specific antibody increases along time and reaches a plateau after the mean duration time of recent infection. Enzyme immunoassay was performed to test the avidity of HIV-specific antibody, and the ODn value of test results can represent the avidity level. The recent infection is classified when ODn value is lower than the cut-off value, and the long-term infection is classified when ODn value is higher than the cut-off value.

Previous geographic studies of HIV infection have usually used data on prevalence in certain areas or the number of newly diagnosed HIV cases to identify hot-spot areas of transmission (4, 5). However, people infected with HIV commonly have no specific symptoms and are not aware of their infection, and once diagnosed, they may have been infected for years. Antiretroviral therapy (ART) can effectively inhibit the replication of the virus and extend the life of people infected with HIV. Therefore, the prevalence data reflect the cumulative effect of HIV infection in the population, which may have a time-lag effect and do not accurately reflect the features of transmission currently occurring. Instead, recent HIV infection, defined as recently acquired HIV infection within a year, is a direct indicator of current epidemic (6). There are several laboratory methods for testing recent HIV infection, which can distinguish between people recently infected with HIV within about 4 months from those infected for long-term (7). Currently, the most widely used laboratory assay for recent infection is the limiting antigen avidity assay (LAg-EIA), which classifies recent HIV infection from long-term HIV infection, according to a principle that the avidity of HIV-specific antibodies is increasing along the time after primary infection and reaching to a plateau later on. Therefore, the test result of antibody avidity lower than a cut-off value can indicate an early stage of HIV infection (7) (Figure 1). In comparison to newly diagnosed HIV cases that may have been infected for many years, recently infected HIV cases are better indicators of new infections and provide more accurate information on the current HIV epidemic. However, only a few studies have conducted geospatial analysis of recent HIV infections (8). Based on the geographic information of recently infected HIV cases, new geospatial analysis measures can be developed to identify areas where the number of new HIV infections is increasing, thus accurately identifying hot-spot areas where transmission is occurring.

The spatial distribution of the HIV epidemic in China is uneven (9). Although the overall HIV prevalence in China is low, local prevalence is high in some areas (10). In Liangshan Yi autonomous prefecture located in Southwest China, there are four neighboring key counties, Butuo, Zhaojue, Yuexi and Meigu, which are areas most severely affected by HIV, with a high HIV prevalence exceeding 1% (11). According to consecutive cross-sectional surveys conducted in Liangshan Prefecture from 2009 to 2018, the major circulating HIV subtype is CRF07_BC, which infected more than 90% of HIV cases in the area (12).

In this study, using the geographic information of HIV cases identified from a whole population physical examination in the four key counties during November 2017 to June 2018, we explored multiple geospatial analytic measures to quantitatively compare the geographic distribution of recently infected and long-term infected cases, and developed an effective strategy to identify the hot-spot areas where new HIV infection is increasing. To our knowledge, no similar studies have been conducted to quantitatively compare the geographical distribution of recent and long-term HIV infections. Our study provides important lessons for identifying hot-spot areas with expending new infection in other regions suffering from a severe HIV burden.

## Methods

### Study Population

Under government-funded public health services, a whole population physical examination was carried out in township health centers in each town of the four key counties from November 2017 to June 2018. For individuals participating in this physical examination, an informed HIV antibody rapid tests (Wondfo Biotech Co., Ltd. Guangzhou, China) was included to expand the coverage of HIV testing services. Over 95% of residents in the four key counties received an on-site HIV antibody test. Individuals with positive HIV antibody screening results but not in the national database of HIV/AIDS cases further had western blot tests (MP biomedicals Asia Pacific, Singapore) to confirm their HIV infection. All tests were performed following manufacturers' instruction manuals.

### Identification of Recently Infected HIV Cases

To identify recently infected HIV cases from newly diagnosed cases, limiting antigen avidity enzyme immunoassay (LAg-EIA assay, Kinghawk Pharmaceutical Co., Ltd, Beijing, China) for recent HIV infection with good credibility was used to classify recent infection from long-term infection (6). As the manufacturer's instruction manual, the mean duration of recent infection (MDRI) is 130 days, and the false recent rate (FRR) is 2.3% for application in China.

In addition, HIV testing history with a known negative HIV testing result within 6 months prior to the positive HIV testing result during the period of physical examination was also regarded as a recent infection case.

### Demographic and Geographic Information

Demographic information of newly diagnosed HIV cases, including gender, age, marital status, educational attainment, and residential address, were extracted from the national HIV/AIDS database. Geographic data, including altitude and roads for mapping were collected from the National Catalog Service for Geographic Information (http://www.webmap.cn).

### Spatial Analysis

All maps were created using ArcGIS v10.6 (ESRI, Redlands, CA, USA) software. Maps of recent and long-term infection cases were constructed using village midpoint coordinates extracted from resident address information. The kernel density of recent and long-term infection cases was calculated using ArcGIS to visually display the density of cases in the indicated area.

### Statistical Analysis

Statistical analyses were performed using SPSS software (version 23.0; SPSS Inc. Chicago, IL). Categorical variables were compared using Fisher exact tests. All tests were two-tailed, and a *p* < 0.05 was considered statistically significant. Ordinary least squares regression (OLSR) was performed to compare the geographical pattern of recent infection and long-term infection. In OLSR analysis, we set kernel density of recent infection as the response variable and kernel density of long-term infection as the explanatory variable. Residuals of OLSR were calculated to show the difference between recent infection and long-term infection. We defined residuals >2 standard deviation (SD) as an obvious increase of recent infection and residuals ranging between 1 and 2 SD as a slight increase. Similarly, residuals < −2 SD was defined as an obvious decrease of recent infection, and residuals ranging between −2 SD and −1 SD as a slight decrease. Residuals ranging between −1 SD and 1 SD were considered to have no difference. The residuals were visualized on a map using ArcGIS. In addition, Geodetector (http://www.geodetector.cn) was used to perform statistical comparison, which used a *q* value ranging between 0 and 1 to display asymmetry (13, 14).

## Results

### Demographic Characteristics of Newly Diagnosed HIV Cases

Among a total of 5,555 newly diagnosed HIV cases, 5,196 (93.6%, 5,196/5,555) cases have full demographic information, and 217 (4.2%, 217/5,196) cases were classified as recently infected cases. We compared the demographic characteristics between recent and long-term infected cases (Table 1). Significant differences were seen in gender, age group, and transmission routes (*p* < 0.01). Females accounted for 57.6% (125/217) of recently infected cases, significantly higher than 42.5% (2,115/4,979) in long-term infected cases (*p* < 0.001). The age group of 15–29 and 30–44 years accounted for 31.8% (69/217) and 47.5% (103/217), respectively, in recently infected cases, while the proportions of these two age groups were 29.2 and 43.3% in long-term infected cases. Infection through heterosexual transmission accounted for 76.5% (166/217) of recently infected cases, while this proportion was just 58.5% (2,913/4,979) in long-term infected cases. These findings suggest that the newly infected individuals were more likely to be female, in young or middle age, and infected through heterosexual contact.

**Table 1:** Demographic characteristics of included individuals.

	**Recent infection**	**Long-term infection**	***P***
	**(217)**	**(4,979)**	
**Gender**
Male	92 (42.4%)	2,864 (57.5%)	<0.001
Female	125 (57.6%)	2,115 (42.5%)	
**Age**
1.5–14	15 (6.9%)	743 (14.9%)	0.006
15–29	69 (31.8%)	1,452 (29.2%)	
30–44	103 (47.5%)	2,158 (43.3%)	
≥45	30 (13.8%)	626 (12.6%)	
**Marital status**
Single	56 (25.8%)	1,427 (28.7%)	0.318
Married	135 (62.2%)	3,088 (62.0%)	
Divorced/widowed	23 (10.6%)	429 (8.6%)	
Unknown	3 (1.4%)	35 (0.7%)	
**Educational attainment**
No schooling	143 (65.9%)	3,571 (71.7%)	0.158
Primary school	66 (30.4%)	1,241 (24.9%)	
Middle school and above	8 (3.7%)	167 (3.4%)	
**Transmission routes**
Heterosexual contact	166 (76.5%)	2,913 (58.5%)	<0.001
Intravenous drug injection	36 (16.6%)	1,207 (24.2%)	
Others	15 (6.9%)	859 (17.3%)	

### Geographic Features of Newly Diagnosed HIV Cases

**Figure 2:**
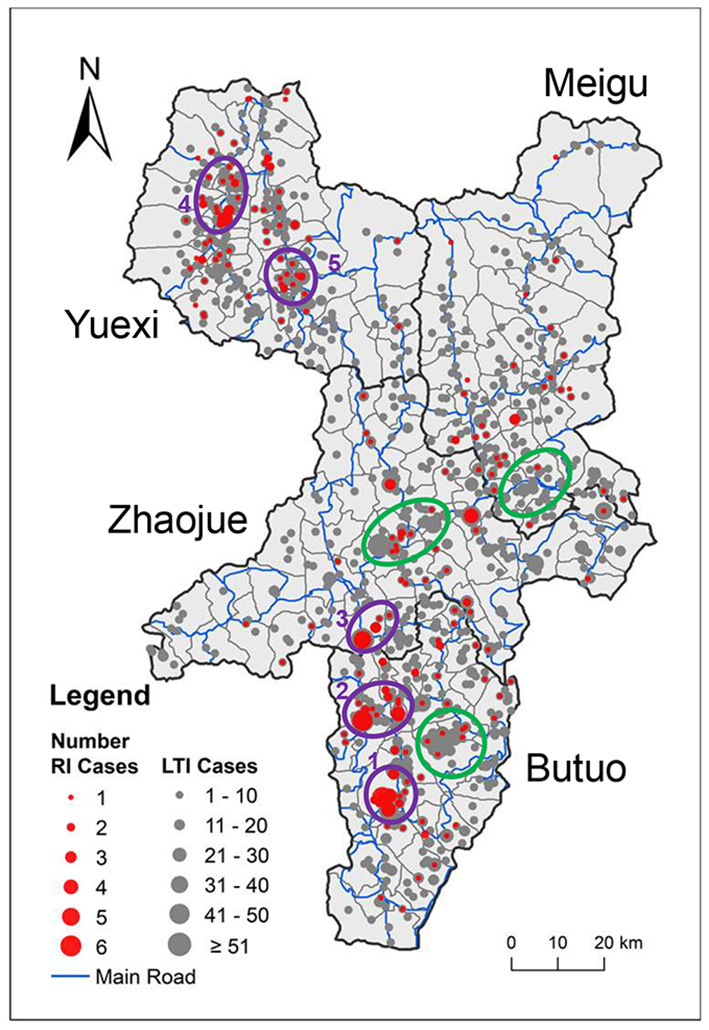
Geographic distribution of newly diagnosed HIV cases. The locations of newly diagnosed HIV cases were mapped onto the administrative map of the four key counties. The size of the circle indicates the number of cases, while red and gray indicate recently infected and long-term infected HIV cases, respectively. County borders are indicated by black lines. Town borders are indicated by gray lines, and main roads are indicated by blue lines. Purple rings 1–5 denote spatial clusters of recently infected HIV cases. Green rings denote clusters of long-term infected cases that overlap with only a few recently infected HIV cases.

The geographic location of the 5,555 newly diagnosed HIV cases was mapped. To display the relative increase of new HIV cases compared to existing HIV cases, we labeled 246 recently infected cases and 5,309 long-term infected cases by red circles and gray circles, separately, so that the spatial distribution between these two populations could be visually compared (Figure 2). The ratio of the size of red circles to the size of gray circles indicates the proportion of recently infected cases to long-term infected cases.

In a whole, the geographic location of HIV cases in the four key counties showed an uneven distribution along main roads and clustering at road intersections. It is noteworthy that some clusters of recently infected cases overlapped with clusters of long-term infected cases, such as cluster 2 and cluster 5, which suggests that the clustering of newly acquired HIV cases might correlated with the high number of existing cases in this area. However, some clusters of recently infected cases were located in areas where not many long-term infected cases were located, such as cluster 1, which suggests a recent growth of new HIV cases in areas with few existing HIV infection and needs closer attention. On the map, there are some clusters of long-term infected cases that overlapped with only a few recently infected HIV cases, indicated by green rings, which suggests that these areas with high number of existing HIV cases did not acquire many new HIV cases recently.

### Quantitatively Comparing the Spatial Distribution Pattern of Recent and Long-Term Infection

**Figure 3:**
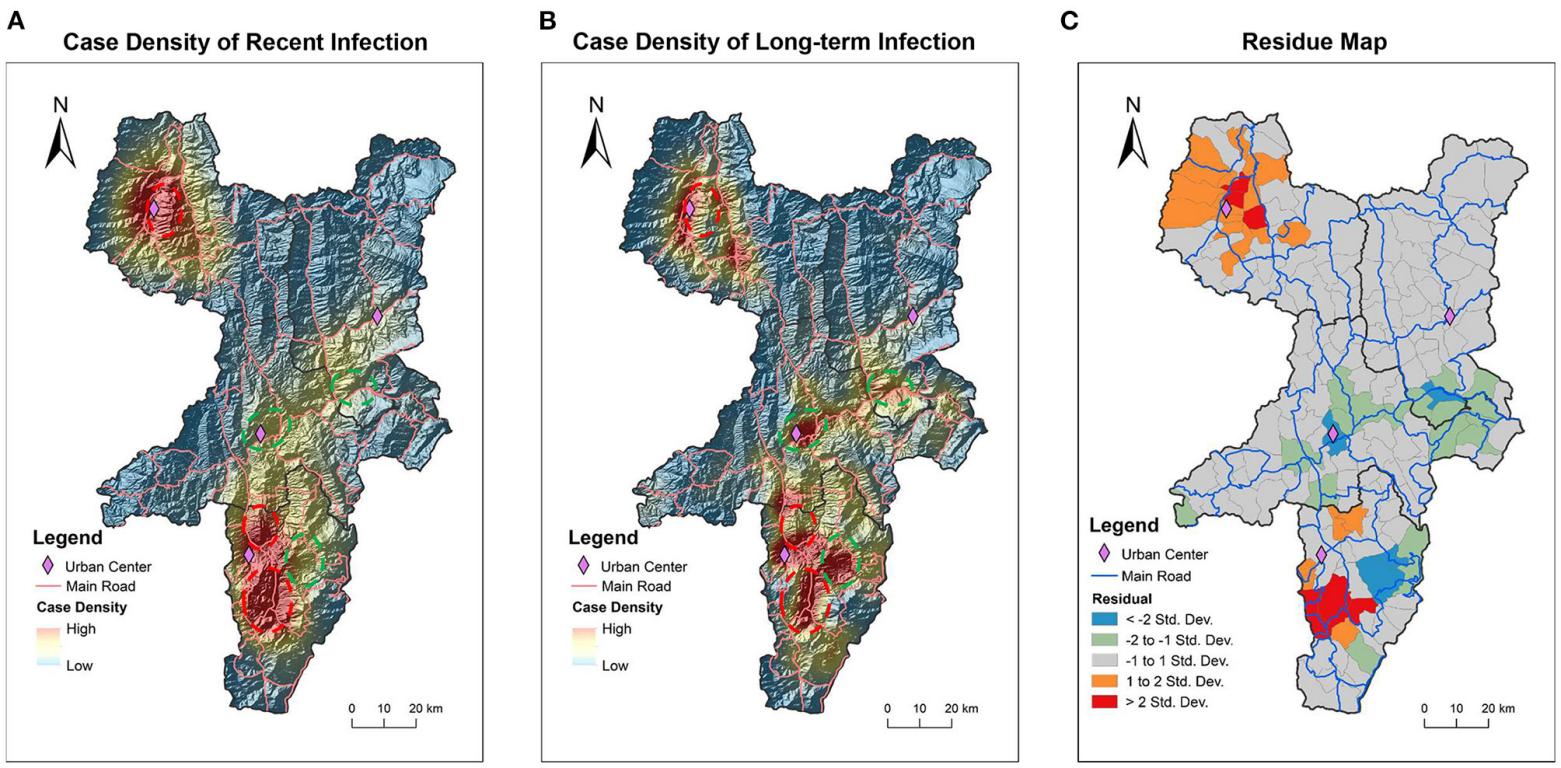
Difference in geographic distribution patterns of recent and long-term infection cases. Kernel density of recently infected HIV cases **(A)** and long-term infected HIV cases **(B)** was calculated and mapped on topographic maps. The value of density is displayed through a gradient colormap and the altitude terrain is shown through shading. Dashed red rings encircle the areas showing obviously higher kernel density of recent infection than long-tern infection. Dashed green rings encircle the areas showing obviously lower kernel density of recent infection than long-tern infection. The statistically calculated residuals from OLSR were mapped to visualize the spatial variation between recent and long-term infection **(C)**. The areas indicated by red and orange have higher kernel density of recent infection, while the areas indicated by blue and green colors have lower kernel density of recent infection. The areas in gray have no difference in kernel density between recent and long-term infection. County borders are indicated by black lines. Town borders are indicated by gray lines, and main roads are indicated by blue lines.

In addition to descriptive analysis, in order to quantitatively compare the spatial distribution patterns of recent and long-term infection, the kernel density of recent and long-term infection was calculated and shown on a topographic map (Figures 3A,B). It can be seen visually and clearly that some areas have high kernel density of long-term infection with low kernel density of recent infection as indicated by dashed green circles. And in contrast, some areas have low kernel density of long-term infection with high kernel density of recent infection as indicated by dashed red circles.

Based on the calculated kernel density of recent and long-term infection, the OLSR and Geodetector analysis were further used to statistically analyze the spatial variation between the geographic distribution of recent and long-term infection (Table 2). In OLSR analysis, the calculated asymmetry for all four key counties is 0.30 (*p* < 0.01), which means 30% geographical variations of recent infection cannot be explained by long-term infection. In Geodetector analysis, the asymmetry for all four key counties is 0.31 (*p* < 0.01), slightly higher than the results from OLSR analysis. In both analytic measures, Butuo showed the highest asymmetry between geographic distribution of recent and long-term infection. These statistical analyses of geospatial- asymmetry suggest that the spatial distribution of new infection had features that were different from previously existing infection, especially in Butuo.

**Table 2:** Asymmetry of spatial distribution between recent and long-term infection cases.

	**All**	**Butuo**	**Meigu**	**Yuexi**	**Zhaojue**
Geodetector *q* value	0.31	0.47	0.39	0.20	0.24
OLSR *R*^2^	0.30	0.44	0.50	0.15	0.27

*OLSR, Ordinary least squares regression*.

To further showcase the geospatial-heterogeneous areas with significant difference in geographic distribution patterns between recent infection and long-term infection, the residuals calculated from OLSR were visually mapped (Figure 3C). Among 151 townships in four key counties, 107 (70.9%, 107/151) townships showed no difference in geographic distribution for recent and long-term infection as indicated by the gray areas. There were 23 townships that showed increased recent infection with 6 townships (4.0%, 6/151) showing obvious increases as indicated by red and 17 townships (11.3%, 17/151) showing slight increases as indicated by orange. In contrast, there were 21 townships that showed decline in recent infection with 4 townships (2.6%, 4/151) showing obvious decline as indicated by blue areas and 17 townships (11.3%, 17/151) showing slight decline as indicated by green areas. It is worth noting that the geospatial-heterogeneous areas shown by residual map analysis are similar to the areas that we observed differences in through visual comparison of the two kernel density maps (Figures 3A,B). Thus, we are assured that the residual map can quantitatively display areas with differences in the spatial distribution of recent and long-term HIV infection. And 23 townships were identified as hot-spot areas showing increased recent HIV infection in comparison to existing long-term infection.

## Discussion

The difference in the spatial distribution between recent and long-term infections may indicate a changing HIV epidemic in the area, and the hot-spot areas in growth of new HIV infection will incentivize focus of intervention efforts there. Our study first distinguished recently infected cases from long-term infected cases by conducting laboratory HIV recency assay on all newly diagnosed HIV cases which were found during a cross-sectional whole population physical examination in 4 neighboring counties with high HIV prevalence in Southwest China. We then investigated the geographic distribution features of recent and long-term HIV cases and developed a geospatial analytic strategy to quantitatively identify hot-spot areas of growing new HIV infection. Using this strategy, we identified 6 townships (4.0%, 6/151) with an obvious increase of new infection and 17 townships (11.3%, 17/151) with a slight increase of new infection. These areas with an increase in new HIV infections may have a higher risk of HIV transmission. Therefore, the results of our study have important implications for planning intervention programs to control the growing epidemic in these hot-spot areas.

Recently, testing result from HIV recency assay has been used in conjunction with geographic information to identify clusters of recent HIV infection, and this strategy has been advocated for in effectively triggering public health responses in varied scopes. In 2019, the United States President's Emergency Plan for AIDS Relief (PEPFAR) funded 16 countries nearing the 90–90–90 targets to initiate the TRACE initiative (Tracking with Recency Assays to Control the Epidemic) (15). HIV recency testing is not only applied to cross-sectional household surveys for estimating HIV incidence, but also offered to persons with new HIV diagnoses in a daily surveillance system (16, 17). In the future, integrating recency testing data with automated analyses on a visualization map could facilitate real-time monitoring of hot-spot areas of new HIV infection in the region. These innovative uses of HIV recency testing data can guide resource allocation for targeted intervention measures to control the epidemic on a local as well as national level.

In this study, we explored several geographic analysis measures in hopes of accurately identifying hot-spot areas of growing new HIV infection in the four key counties. By visually comparing the geographic location of recent and long-term infected cases on the same map, we were able to quickly identify areas with increased new infection or decreased new infection. To rigorously pinpoint hot-spot areas of increasing new HIV infection, we also used spatial statistical analyses to quantify the heterogeneity in spatial distribution between recent infection and long-term infection. Further, we mapped the residuals of OLSR to visualize the geographic areas with significant difference between recent and long-term infection. Through these statistical spatial analysis, 23 townships with increase in new HIV infection were quantitatively identified in Butuo and Yuexi.

HIV recent infections reflect the concurrent HIV transmission characteristics, and which can be controlled by combining public health intervention measures (3). As shown in Figure 3C, HIV recent infection cases showed significant increase in some urban centers. There could be many factors affecting the clustering of HIV recent infections, such as local HIV burden, population density, traffic convenience, social culture, public health policies, etc. (18, 19). For areas with significant increase of recent infections, proper public health policies should be in place to curb the ongoing spread of HIV.

Recent infection testing algorithm (RITA) integrates more information, such as history of HIV infection, ART information, viral load, CD4 counts, which can reduce the misclassification of recently infected HIV cases and is recommended by WHO guidelines (6). In our study, we excluded known long-term HIV cases based on information from the national HIV/AIDS database and performed LAg-EIA assay on newly diagnosed HIV cases which had not received ART. Therefore, the misclassification rate of recent infection in this study is low. In four key counties of Liangshan, at the same time as the survey was conducted, we determined the local FRR value of LAg-EIA assay using specimens from 879 local long-term HIV cases, and 13 specimens among them were falsely classified as recent infection, which estimates local FRR as 1.48% (95%CI: 1.45–1.51%). This study mainly focused on recently infected HIV cases and did not involve the estimation of HIV incidence. Considering the low FRR, the bias caused by misclassification of recent infection is low and will not impact the main results of this study.

Our study was subjected to some limitations. Due to outmigration for work, some residents in the four counties could not participate in the physical examination and the demographic information of some HIV cases were missing, which may have impacted the analysis results. However, the geospatial analytic strategy we have developed is suitable for cross-sectional studies that do not require historic data.

In a whole, our new strategy features a combination of HIV recency testing results with geographic information, using the quantitative analysis to identify hot-spot areas where the geospatial patterns between recent and long-term infection showed significant difference. This new strategy helps to accurately pinpoint hot-spot areas where new HIV infections are growing. By identifying hot-spot areas with increasing trends of new HIV infection, our study provides a new perspective for describing the HIV epidemic. In particular, we hope that our analyses and experiences can be applied to other regions suffering with high HIV burdens in China as well as globally to provide precise and effective geo-guided interventions to help curb the spread of the HIV epidemic.

## Data Availability Statement

The data that support the findings of this study are available from the corresponding author upon reasonable request.

## Ethics Statement

The studies involving human participants were reviewed and approved by the Institutional Review Board of the National Centre for AIDS/STD Control and Prevention, Chinese Centre for Disease Control and Prevention. Written informed consent from the participants' legal guardian/next of kin was not required to participate in this study in accordance with the national legislation and the institutional requirements.

## Author Contributions

CJ and ZL proposed the research question and designed the study. CJ, QZ, CJK, CX, PL, and MC wrote the report. QZ and CX performed the epidemiology and geographic analyses. CJK, SL, GY, JW, and LX contributed to laboratory experiments and epidemiology data collection. PG provided expert knowledge. All authors have seen and contributed to the final version of the report.

## Conflict of Interest

The authors declare that the research was conducted in the absence of any commercial or financial relationships that could be construed as a potential conflict of interest.
